# The mediating role of life satisfaction in the relationship between physical exercise and depressive symptoms among middle-aged and elderly individuals

**DOI:** 10.3389/fpubh.2025.1511509

**Published:** 2025-02-13

**Authors:** Ting Yang, Xinxin Li, Xinyu Wang, Jingjie Zhou, Wei Chen

**Affiliations:** ^1^The Affiliated Xuzhou Rehabilitation Hospital of Xuzhou Medical University, Xuzhou Medical University, Xuzhou, China; ^2^The Xuzhou Clinical College of Xuzhou Medical University, Xuzhou Medical University, Xuzhou, China

**Keywords:** physical exercise, life satisfaction, depression, mediating effect, Chinese

## Abstract

**Objective:**

To investigate the mediating effect of life satisfaction between physical exercise behavior and depressive symptoms among middle-aged and elderly individuals, providing a reference for improving depressive conditions in this demographic group.

**Methods:**

Data from 11,101 middle-aged and elderly individuals from the 2020 China Family Panel Studies (CFPS) were collected. STATA 17.0 was used for data cleaning, organization, and statistical analysis which includes univariate analysis, stepwise regression analysis, and mediation effect testing.

**Results:**

Among 11,101 individuals aged 45 years and above, 2,272 participated in physical exercise, accounting for 20.47%; 2,052 exhibited depressive symptoms, representing 18.48%. Physical exercise was positively correlated with life satisfaction and negatively correlated with depression. Depression scores also showed a negative correlation with life satisfaction. According to the results of the mediation effect study, life satisfaction accounted for 16.60% of the overall effect and had a mediating effect value of −0.099 on depression symptoms.

**Conclusion:**

Physical exercise and life satisfaction are factors influencing depressive symptoms, and life satisfaction acts as a partial mediator between physical exercise participation and depression among middle-aged and elderly adults.

## 1 Introduction

Amidst notable shifts in China's demographic composition, there has been a marked and rapid rise in the percentage of the elderly within the population. According to census data provided by the National Bureau of Statistics, the proportion of the population aged 65 and above increased from 7% in 2000 to 13.5% in 2020, and the proportion of individuals aged 60 and above was 18.7% in 2020. Based on the population structure, it can be inferred that China has become an aging society and has reached a stage of deep aging (1). Against this social backdrop, the concept of healthy aging was proposed. The concept of healthy aging was put forward by the World Health Organization in 1987 as a process that sustains and develops the functional capabilities contributing to the wellbeing of the elderly. It aims to enhance the functional health of older adults, improve their quality of life, and foster the healthy development of societies with aging populations. Physical and psychological health of individuals in middle-aged and advanced-age groups is particularly noteworthy. The “Healthy China 2030” Planning Outline (2) advocates for nationwide participation in physical exercise, aiming to increase the prevalence of physical activity, promote healthy aging, and build a solid foundation for the sustainable development of the nation. As a psychological and social activity, physical exercise holds significant social functions. It can improve cognitive abilities and the brain's reaction speed. Not only can it prevent various chronic diseases, but it can also enhance psychological wellbeing, subjective happiness, and life satisfaction. Physical activity is crucial for enhancing residents' overall health and shaping a positive social spirit (3, 4).

Depression, a global public health challenge, has become a prevalent mental health condition affecting middle-aged and older adults (5, 6). According to scholarly assessments, estimated 350 million individuals worldwide are living with depression, with a prevalence of 6.9% among adults in China (7, 8).Some studies indicate the potential for depression to become the predominant source of the global health burden by 2030 (9–11). The typical manifestations of depression include low mood, pessimistic thinking, and slowed cognitive function. These symptoms not only increase the risk of self-harm, suicide, and cognitive impairment but may also lead to physical functional limitations, affecting the quality of life and daily activity capabilities of patients, thereby exacerbating the burden on family economics and social resources (12). The widespread outbreak of COVID-19 has deeply affected daily life, diminished social engagement, heightened financial strain, and more frequent encounters with distressing news and emotions, thereby substantially increasing the prevalence of anxiety and depression across the general public (13).

Life satisfaction represents an individual's holistic assessment of their life conditions over a significant timeframe, encompassing cognitive judgments about the overall quality of their experiences based on subjectively set standards (14). It holds significant importance for the health, wellbeing, and development of individuals. It serves as a measure of happiness index and is an essential component in achieving healthy aging and implementing the Healthy China strategy. When individuals have better social support, economic status, and social security, their life satisfaction tends to be higher.

The influencing factors of depression have been extensively studied. Previous research has indicated that physical exercise has antidepressant effects and can improve the psychosocial functions of older adults (15). The previous research indicates that life satisfaction emerges as the most potent inverse indicator of depressive tendencies (16). However, previous studies have not indicated the mediating role of life satisfaction in the impact of physical exercise on depressive symptoms.

Concerning the advancement of the Healthy China strategy and the virtue of physical exercise on both physical and psychological health, along with this research attempts to investigate the role of life satisfaction in modulating the relationship between exercise and depression symptoms in middle-aged and older people, in hopes of providing a scientific basis for improving their psychological health status and reducing the incidence of depression. To this end, this study utilizes 2020 follow-up data from the China Family Panel Studies (CFPS) project, with the research findings indicated below.

## 2 Materials and methods

### 2.1 Data source

This information originated from the 2020 China Family Panel Studies (CFPS) data repository (online data access URL: http://www.isss.pku.edu.cn/cfps/) (17). The study received ethical clearance from Peking University Biomedical Ethics Committee (IRB00001052-14010). The CFPS project team obtained written informed consent from participants before data collection. Prior to data analysis, identifying information about the participants was anonymized. This survey is the sixth round of a national investigation aimed at reflecting changes in Chinese society. The project employed a systematic sampling method that is representative of the demographic traits of 95% of the Chinese population. The objective of the 2020 follow-up survey was to include 16,000 households, encompassing all members of the families within the selected households. This study selected relevant data from individuals aged 45 years and above to analyze how physical exercise affects depressive symptoms. The collected variables included age, sex, place of residence, education level, marital status, physical exercise, self-rated health, life satisfaction, smoking status, chronic disease conditions, Internet usage, participation in medical insurance, and depression status. Following the handling of invalid data in similar studies (18), records with key variable results labeled as “don't know,” “refuse to answer,” or “not applicable” were excluded, leaving 11,101 valid data entries.

### 2.2 Variables selection

#### 2.2.1 Control variables

The study's control variables included age, gender, place of residence, education, marital status, smoking status, chronic disease conditions, self-rated health status, Internet usage status, and medical insurance participation status.

#### 2.2.2 Independent variable

The study's independent variable was physical exercise, with the question being, “Excluding cycling or walking solely for commuting purposes, what is the frequency of your engagement in sports, fitness, or recreational activities during the past year? How long do you exercise every time?” (19). The criteria for classifying participation in physical exercise in this study are based on existing research recommendations regarding the dosage of exercise intervention for depression treatment, which suggests that engaging in moderate-intensity physical exercise for at least 30 min on three or more days a week is the ideal level of intensity for alleviating depression (20). Participation in physical exercise was defined as engaging in sports activities three to four or more times per week, with each session lasting over 30 min; otherwise, it was considered non-participation in physical exercise.

#### 2.2.3 Mediator variable

Life satisfaction was the mediator variable. The question was, “How content are you with your life, ranging from one to five, where one is highly dissatisfied and five is very satisfied?”

#### 2.2.4 Dependent variable

The dependent variable was depression. The study utilizes an abridged 8-item version of the Center for Epidemiologic Studies Depression Scale (CES-D), encompassing eight items that pertain to the following aspects: “I was depressed,” “I thought that everything required effort,” “ I experienced poor sleep,” “ I experienced a sense of happiness,” “ I experienced a sense of alone,” “I felt glad in life,” “I felt life was not worth living,” and “I felt sad.” The available choices are as follows: “Infrequently (< 1 day),” “Occasionally (1–2 days),” “Frequently (3–4 days),” and “Almost always (5–7 days).” The scores are assigned values that range from 1 to 4 on an individual basis. An elevated overall score suggests greater severity of depressive symptoms. This study adopted the critical point for depression scores based on the overall cut-off score recommended by Radloff, which is the 80th percentile of the scale score (17 points) (21).

### 2.3 Statistical analysis

Statistical processing was carried out using Stata 17.0 for univariate analysis, Spearman's correlation analysis, three-step regression analysis, bootstrap mediation effect testing and subgroup analysis. A two-sided significance level of 0.05 was set.

## 3 Results

### 3.1 Basic characteristics of the research subjects and difference testing

This research encompassed 11,101 participants who were classified as middle-aged and elderly, comprising 5,487 females (49.43%) and 5,614 males (50.57%); Age distribution: 6,378 individuals aged 45–60 (57.45%) and 4,723 individuals aged over 60 (42.55%); Place of residence: 5,717 in rural areas (51.50%) and 5,384 in urban areas (48.50%); Education level: 6,010 with primary school or below (54.14%), 3,135 with junior high school (28.24%), 1,435 with high school (12.93%), and 521 with college or above (4.69%); Marital status: 1,277 divorced/unmarried/widowed (11.50%) and 9,824 married/cohabitating (88.50%); smoking status: 7,874 non-smokers (70.93%) and 3,227 smokers (29.07%); Chronic disease status: 8,585 without (77.34%) and 2,516 with (22.66%); Internet usage: 4,568 non-users (41.15%) and 6,533 users (58.85%); Medical insurance participation: 840 without (7.57%) and 10,261 with (92.43%). Additionally, 2,272 individuals (20.47%) participated in physical exercise and 2,052 individuals (18.48%) exhibited depression. The outcomes indicated that depressive symptoms significantly differed across various demographics, including gender, place of residence, education level, marital status, health status, smoking, chronic disease, Internet usage, and medical insurance participation (*P* < 0.001) (Table 1).

**Table 1:** Basic information of the research subjects and difference testing.

**Variable**	***n* (%)**	***n* of depression (%)**	**χ^2^/*Z***	***P***
**Age**				
45–60	6,378 (57.45)	1,149 (18.02)		
60~	4,723 (42.55)	903 (19.12)	2.196	0.138
**Gender**				
female	5,487 (49.43)	1,212 (22.09)	93.515	< 0.001
male	5,614 (50.57)	840 (14.96)		
**Place of residence**				
rural	5,717 (51.50)	1,272 (22.25)	110.869	< 0.001
urban	5,384 (48.50)	780 (14.49)		
**Education level**				
Primary school or below	6,010 (54.14)	1,376 (22.90)	175.590	< 0.001
Junior high school	3,135 (28.24)	449 (14.32)		
Senior high school	1,435 (12.93)	172 (11.99)		
College or above	521 (4.69)	55 (10.56)		
**Marital status**				
Divorced/Single/Widowed	1,277 (11.50)	400 (31.32)	157.850	< 0.001
Married/Cohabitating	9,824 (88.50)	1,652 (16.82)		
**Health status**				
Very healthy	1,363 (12.28)	139 (10.20)	817.029	< 0.001
Quite healthy	1,326 (11.94)	131 (9.88)		
Fairly healthy	4,436 (39.96)	576 (12.98)		
Average	1,610 (14.50)	306 (19.01)		
Unhealthy	2,366 (21.31)	900 (38.04)		
**Life satisfaction**				
Very dissatisfied	155 (1.40)	92 (59.35)	695.237	< 0.001
Dissatisfied	302 (2.72)	165 (54.64)		
Neutral/average	2,324 (20.94)	647 (27.84)		
Satisfied	3,444 (31.02)	515 (14.95)		
Very satisfied	4,876 (43.92)	633 (12.98)		
**Smoking status**				
No	7,874 (70.93)	1,521 (19.32)	12.441	< 0.001
Yes	3,227 (29.07)	531 (16.45)		
**Chronic disease status**				
No	8,585 (77.34)	1,366 (15.91)	166.470	< 0.001
Yes	2,516 (22.66)	686 (27.27)		
**Physical exercise status**				
No	8,829 (79.53)	1,762 (19.96)	62.045	< 0.001
Yes	2,272 (20.47)	290 (12.76)		
**Internet usage status**				
No	4,568 (41.15)	651 (14.25)	92.326	< 0.001
Yes	6,533 (58.85)	1,401 (21.44)		
**Medical insurance participation status**				
No	840 (7.57)	207 (24.64)	22.871	< 0.001
Yes	10,261 (92.43)	1,845 (17.98)		

### 3.2 Correlation analysis of physical exercise, depressive status, and life satisfaction

Spearman's correlation analysis revealed substantial statistical evidence supporting an inverse association between physical exercise and depressive status (*r* = −0.100, *P* < 0.001), a significant positive association between physical exercise and life satisfaction (*r* = 0.016, *P* < 0.05), and a statistically significant negative association between life satisfaction and depressive status (*r* = −0.277, *P* < 0.001) (Table 2).

**Table 2:** Correlation analysis of physical exercise, life satisfaction and depressive symptoms.

**Variable**	**Physical exercise**	**Depression status**	**Life satisfaction**
Physical exercise	1.000		
Depression status	−0.100^***^	1.000	
Life satisfaction	0.016^*^	−0.277^***^	1.000

^*^p < 0.05, ^**^p < 0.01, ^***^p < 0.001.

### 3.3 Analysis of the mediating effect of life satisfaction between physical exercise and depressive status

**Figure 1:**
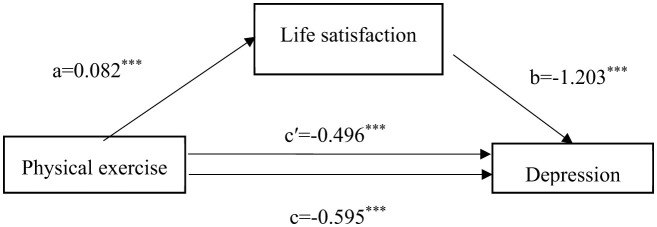
Mediation effect model of life satisfaction between physical exercise and depression. ^*^P < 0.05, ^**^P < 0.01, ^***^P < 0.001; a represents the effect value of physical exercise on life satisfaction, b represents the effect value of life satisfaction on depressive status, c represents the total effect value of physical exercise on depressive status, and c' represents the direct effect value of physical exercise on depressive status.

This study employed the causal step regression method (22, 23) to test for mediation effects. In the first step, with physical exercise as the independent variable and depressive status as the dependent variable, a regression analysis was performed, denoted as Model 1. The outcomes indicated that physical exercise had an impact on depressive status (c value, t = −5.98, *P* < 0.001) (Table 3). In the second step, regression analysis was performed with physical exercise as the independent variable and life satisfaction as the dependent variable, revealing that physical exercise affected life satisfaction (a value, t = 3.72, *P* < 0.001), which is referred to as Model 2. In the third step, based on Model 1, the mediation variable of life satisfaction was included in the regression analysis, and the results indicated that both physical exercise and life satisfaction affected depressive status (c' value, t = −5.17; b value, t = −29.11). Figure 1 presents the model after establishing the mediating effect, showing that the total effect of physical exercise on depressive status was −0.595, and ab and c' had the same sign, with |c'| < |c|, suggesting that life satisfaction had a partial mediation effect between physical exercise and depressive status. Table 4 shows that the 95% confidence interval (CI) of the partial mediating effect of life satisfaction did not include 0, which was statistically significant, and the direct effect was also significant. This indicates that life satisfaction has a partial mediating effect between physical exercise and depressive status, with a mediation effect of −0.099, total effect of −0.595, and mediation proportion of 16.60% (Figure 1, Tables 3, 4).

**Table 3:** Regression analysis results of physical exercise and life satisfaction on depressive symptoms in middle-aged and elderly adults.

**Independent variable**	**Model1**		**Model2**		**Model3**	
	β	***t***	β	***t***	β	***t***
Physical exercise	−0.595^***^	−5.98	0.082^***^	3.72	−0.496^***^	−5.17
Life satisfaction					−1.203^***^	−29.11
Constant	13.461^***^	60.19	4.224^**^	84.86	18.54^***^	66.85
R^2^		0.154	0.076		0.214	

^*^P < 0.05, ^**^P < 0.01, ^***^P < 0.001: Model 1 predicts depressive status based on physical exercise; Model 2 predicts depressive status based on life satisfaction; Model 3 predicts depressive status with both physical exercise and life satisfaction as predictors.

**Table 4:** Bootstrap mediation effect test of life satisfaction between physical exercise and depressive symptoms in middle-aged and elderly adults.

	**Coefficient**	**Bootstrap SE**	***P***	**Boot 95%CI**	**Effect size(%)**
Indirect effect	−0.099	0.024	< 0.001	(−0.146, −0.051)	16.60
Direct effect	−0.496	0.087	< 0.001	(−0.667, −0.325)	83.40

### 3.4 Subgroup analysis

The of the subgroup analysis are shown in Table 5. Life satisfaction has a partial mediating effect between physical exercise and depressive symptoms in middle–aged individuals (z = −3.75; indirect effect = −0.122, CI = (−0.185, −0.058), *P* < 0.001; z = −2.42, direct effect = −0.291, CI = (−0.527, −0.055), *p* = 0.016). Significance is observed in both gender and place of residence subgroups (*P* < 0.05). Among them, female (z = −2.29; indirect effect = −0.086, CI = (−0.160, −0.012), *P* = 0.022; z = −3.54, direct effect = −0.456, CI = (−0.709, −0.204), *p* < 0.001); for male (z = −3.31; indirect effect = −0.109, CI = (−0.173, −0.446), *P* = 0.001; z = −4.26, direct effect = −0.542, CI = (−0.791, −0.293), *P* < 0.001); in rural areas (z = −2.40; indirect effect = −0.101, CI = (−0.183, −0.018), *P* = 0.016; z = −3.00, direct effect = −0.470, CI = (−0.776, −0.163), *P* = 0.003); and in urban areas (z = −2.86; indirect effect = −0.093, CI = (−0.157, −0.029), *P* = 0.004; z = −4.97, direct effect = −0.544, CI = (−0.760, −0.330), *P* < 0.001).

**Table 5:** Subgroup analysis of mediation effect.

		**Effect**	**Coefficient**	**Bootstrap SE**	**z**	***P***	**Boot 95%CI**
Age	45–60	Indirect effect	−0.122	0.032	−3.75	< 0.001	(−0.185,−0.058)
		Direct effect	−0.291	0.120	−2.42	0.016	(−0.527,−0.055)
	60~	Indirect effect	−0.716	0.037	−1.95	0.051	(−0.144,0.000)
		Direct effect	−0.703	0.137	−5.14	< 0.001	(−0.971,−0.435)
Gender	Female	Indirect effect	−0.086	0.038	−2.29	0.022	(−0.160,−0.012)
		Direct effect	−0.456	0.129	−3.54	< 0.001	(−0.709,−0.204)
	Male	Indirect effect	−0.109	0.033	−3.31	0.001	(−0.173,−0.446)
		Direct effect	−0.542	0.127	−4.26	< 0.001	(−0.791,−0.293)
Place of residence	Rural	Indirect effect	−0.101	0.042	−2.40	0.016	(−0.183,−0.018)
		Direct effect	−0.470	0.156	−3.00	0.003	(−0.776,−0.163)
	Urban	Indirect effect	−0.093	−0.325	−2.86	0.004	(−0.157,−0.029)
		Direct effect	−0.544	0.110	−4.97	< 0.001	(−0.760,−0.330)

## 4 Discussion

The study indicates that the participation rate in physical exercise was 20.47%, which differs from the results of studies by Li et al. (24), Zhang et al. (25), and Gao et al. (26) and is related to the sample range and timing. The positive screening rate for depression among 11,101 middle-aged and elderly individuals aged 45 and above was 18.84%, which differs from the results of studies by Cai et al. (12), possibly due to the different diagnostic criteria for depression and sampling methods of the selected samples.

The study revealed correlations between physical exercise, life satisfaction, and depression scores among middle-aged and older individuals. Physical exercise and life satisfaction are positively correlated, in line with the findings of MAHER (27) and Wei et al. (28). This may be because physical exercise helps individuals form positive self-concepts, enhance positive self-perceptions, strengthen their perception of future health, promote social interaction and participation, and increase perceived social support, thereby improving life satisfaction (29, 30). Life satisfaction is negatively correlated with depression scores. Long-term high life satisfaction promotes the functional development of this structure, thereby better resisting depressive mood (31). Physical exercise was negatively correlated with depression scores, similar to the results of studies by Wang et al. (32, 33). This may be because physical exercise can bring both short-term and long-term psychological benefits. Short-term psychological benefits can improve mood without depending on the objective state of physical health, representing an individual's evaluative experience (34). Looking at the long-term effects, physical exercise provides both physical and psychological benefits. Exercise strengthens the body and indirectly improves the psychological state. On the other hand, physical exercise promotes the secretion of endorphins and the absorption of excitatory neurotransmitters, enhancing the brain's sources of pleasure (32) thus maintaining a positive emotional experience. At the same time, exercise effectively protects the brain's plasticity and promotes brain health. Appropriate exercise helps to preserve the structural integrity of the hippocampus and white matter, promotes hippocampal regeneration, improves brain neural processing efficiency, and delays cognitive decline, thereby alleviating depressive states (35).

This study indicated that life satisfaction partly mediates the relationship between physical exercise and depressive symptoms, contributing to a mediation effect of −0.099, which represents 16.60% of the overall effect. This suggests that physical exercise can directly alleviate depressive symptoms and indirectly reduce the incidence of depression by enhancing life satisfaction. This mechanism of action can be explained from both physiological and psychological perspectives. As age increases, multiple functions of the central nervous system are affected, leading to reduced stability and flexibility. Long-term physical exercise is beneficial for increasing the weight of the cerebral cortex, delaying the aging of central nervous system functions, accelerating the speed of new conditioned reflex connections, and improving immediate memory (36), executive control (37), processing velocity (38), and attention, thereby enhancing social auxiliary factors such as language fluency and cognitive flexibility (39). Cognitive deficits are a key factor in the decline of social and psychological functions (36) and improvements in cognitive function help address psychological issues arising from aging, thereby improving the subjective evaluation of quality of life (40). Psychologically, this population, characterized by social decline (41),experiences a decrease in social relationships and support as well as a weakening of social roles (42). These declines in social functioning can easily lead to psychological problems. Physical exercise promotes the construction of new social roles and interactions, obtains new social support, alleviates mental health issues related to social emotions, enhances social motivation to participate in social activities, and strengthens individual self-perception (43). When engaging in physical exercise, individuals are less focused on their negative emotions and more focused on the positive emotions generated by exercise (44). Physical exercise helps boost self-confidence and improve self-esteem and self-efficacy (45), thereby improving the assessment of one's quality of life. Individuals with high life satisfaction tend to have higher psychological resilience, which helps them cope with negative emotions (46), and higher life satisfaction provides positive feedback to the nervous system (31), reducing the risk of depressive symptoms. Notably, the mediation effect analysis results demonstrate that life satisfaction has a higher direct effect value on depressive symptoms than the direct effect of physical exercise, suggesting that raising life satisfaction has a greater influence on the reduction of depressive symptoms. The results of the subgroup analysis of the selected variables indicate that the mediating effect has a certain degree of robustness.

Therefore, to promote the psychological wellbeing of middle-aged and older adults, it is essential to encourage this demographic to participate in physical exercise, fully mobilize their enthusiasm for physical activity, and improve their physical health. Simultaneously, it is important to focus on increasing life satisfaction among the middle-aged and elderly, suggesting that government agencies should build a comprehensive elderly health service system (47) and employment security mechanisms for middle-aged individuals, optimize the accessibility of health and senior care services, and strengthen the social security system. Additionally, increasing financial and social resources should be used to promote the establishment of social organizations and enhance social interaction and communication opportunities for middle-aged and elderly individuals, thereby reducing the occurrence of depressive symptoms in this population (48).

## 5 Limitations

This research is a cross-sectional study, which does not allow for a clear determination of causal relationships. Additionally, this study did not differentiate the content of physical exercise, merely categorizing it into binary choices of yes or no based on a standard. The therapeutic effects of different exercise prescriptions require in-depth investigation. Additionally, the methodology section of this study needs to be further strengthened to make the results of the entire study more specific and rigorous.

## 6 Conclusion

In summary, physical exercise and life satisfaction impacted the depressive status of middle-aged and elderly individuals, with life satisfaction playing a partial mediating role. However, the participation rate in physical exercise among middle-aged and elderly individuals in China is relatively low. Given that physical exercise can mitigate the likelihood of developing depression in this demographic, there should be a strong push to publicize the benefits of physical exercise, improve community sports facilities, and promote increased participation in physical activity. Currently, there is a lack of analysis on exercise prescriptions tailored to different population characteristics. Future research should focus on constructing precision exercise prescriptions for different population groups and combining multimodal neuroimaging with various analysis techniques to fully leverage the mitigating effects of physical exercise against depression in middle-aged and elderly individuals. Additionally, attention should be paid to improving the life satisfaction of middle-aged and elderly people, strengthening communication between children and the elderly and perfecting the social security system to ensure that the elderly have joy and purpose in their later years, thereby reducing the incidence of depression among the middle-aged and elderly population.

## Data Availability

Publicly available datasets were analyzed in this study. This data can be found here: http://www.isss.pku.edu.cn/cfps/.
